# Co-production of 11α-hydroxyprogesterone and ethanol using recombinant yeast expressing fungal steroid hydroxylases

**DOI:** 10.1186/s13068-017-0904-z

**Published:** 2017-09-29

**Authors:** Claire M. Hull, Andrew G. S. Warrilow, Nicola J. Rolley, Claire L. Price, Iain S. Donnison, Diane E. Kelly, Steven L. Kelly

**Affiliations:** 1 Institute of Life Science, Swansea University Medical School, Swansea, SA2 8PP Wales UK; 2 Institute of Biological, Environmental & Rural Sciences, Aberystwyth University, Gogerddan, Aberystwyth, Wales SY23 3EE UK

**Keywords:** Bioconversion, Bioethanol, Biorefinery, 11α-Hydroxyprogesterone, Ryegrass, Yeast

## Abstract

**Background:**

Bioethanol production from sustainable sources of biomass that limit effect on food production are needed and in a biorefinery approach co-products are desirable, obtained from both the plant material and from the microbial biomass. Fungal biotransformation of steroids was among the first industrial biotransformations allowing corticosteroid production. In this work, the potential of yeast to produce intermediates needed in corticosteroid production is demonstrated at laboratory scale following bioethanol production from perennial ryegrass juice.

**Results:**

Genes encoding the 11α-steroid hydroxylase enzymes from *Aspergillus ochraceus* (11α-SH^Aoch^) and *Rhizopus oryzae* (CYP509C12) transformed into *Saccharomyces cerevisiae* for heterologous constitutive expression in p425TEF. Both recombinant yeasts (AH22:p11α-SH^Aoch^ and AH22:p509C12) exhibited efficient progesterone bioconversion (on glucose minimal medial containing 300 µM progesterone) producing either 11α-hydroxyprogesterone as the sole metabolite (AH22:p11α-SH^Aoch^) or a 7:1 mixture of 11α-hydroxyprogesterone and 6β-hydroxyprogesterone (AH22:p509C12). Ethanol yields for AH22:p11α-SH^Aoch^ and AH22:p509C12 were comparable resulting in ≥75% conversion of glucose to alcohol. Co-production of bioethanol together with efficient production of the 11-OH intermediate for corticosteroid manufacture was then demonstrated using perennial ryegrass juice. Integration of the 11α-SH^Aoch^ gene into the yeast genome (AH22:11α-SHAoch+K) resulted in a 36% reduction in yield of 11α-hydroxyprogesterone to 174 µmol/L using 300 µM progesterone. However, increasing progesterone concentration to 955 µM and optimizing growth conditions increased 11α-hydroxyprogesterone production to 592 µmol/L product formed.

**Conclusions:**

The progesterone 11α-steroid hydroxylases from *A. ochraceus* and *R. oryzae*, both monooxygenase enzymes of the cytochrome P450 superfamily, have been functionally expressed in *S. cerevisiae*. It appears that these activities in fungi are not associated with a conserved family of cytochromes P450. The activity of the *A. ochraceous* enzyme was important as the specificity of the biotransformation yielded just the 11-OH product needed for corticosteroid production. The data presented demonstrate how recombinant yeast could find application in rural biorefinery processes where co-production of value-added products (11α-hydroxyprogesterone and ethanol) from novel feedstocks is an emergent and attractive possibility.

## Background

Biofuels such as bioethanol are a key focus for sustainability away from fossil fuels and in a biorefinery approach maximum value and product extraction is envisaged. For instance, plant protein or other products may be extracted prior to fermentation of carbohydrates. The microbial biomass also offers routes to co-products. Some of the first industrial biotransformations involved filamentous fungi for production of key intermediates in corticosteroid production that were hard to achieve by chemical synthesis. Ever since the first study reporting microbial 11α-hydroxylation of progesterone using *Rhizopus arrhizus* over 60 years ago [1], there has been sustained scientific and commercial interest in the application of bacteria [2–6] and filamentous fungi [7–9] to produce hydroxylated pharmaceuticals and intermediates. Structural hydroxylations are often necessary to functionalize steroid-based drugs that exhibit higher efficacy than their non-hydroxy analogs [10], and the ability to perform regio- and stereo-specific modifications using whole-cell microbial biocatalysis is invaluable for large-scale steroid manufacturing [11] not least because certain hydroxylations can prove difficult or even impossible to achieve via synthetic chemistry.

**Fig. 1:**
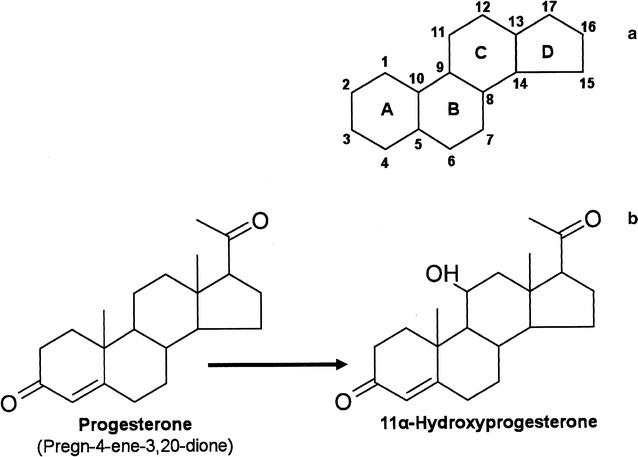
Steroid chemical structure. **a** Gonane, the steroid nucleus comprising three cyclohexane rings (rings A, B, and C) and one cyclopentane (ring D) labeled using the IUPAC-recommended ring-lettering and atom-numbering. **b** Hydroxylation of progesterone to 11α-hydroxyprogesterone

Unlike unicellular yeasts, filamentous fungi (e.g., *Rhizopus*, *Aspergillus,* and *Mucor* spp.) can hydroxylate the sterane skeleton (Fig. 1) at numerous positions (1β, 2β, 4, 5α, 6β, 7α, 7β, 9α, 10β, 11α, 11β, 12α, 12β, 14, 15α, 15β, 16α, 17α, 19, 22, 26) [12]. Alpha and beta hydroxylations at carbon 11 have for more than 50 years been performed commercially using filamentous fungi [11] and are particularly desirable for the synthesis of the glucocorticoid steroids that are commonly prescribed as anti-inflammatory, immunosuppressive, anti-allergic, and contraceptive drugs [13]. However, despite their industrial and biomedical value, species-specific information about 11α-steroid hydroxylases from filamentous fungi and data regarding their prevalence across the fungal kingdom remains limited; instead they are classified very broadly as monooxygenase enzymes of the cytochrome P450 (CYP) superfamily. In fact, sequencing data for just two fungal 11α-steroid hydroxylases from *Aspergillus ochraceus* [14] and *Rhizopus oryzae* [15] are currently available. Following historical neglect, it is hoped that the recent establishment of fungal genome databases and increasing interest in transcriptional data for filamentous species will now permit new biotechnological approaches that will harness the full biosynthetic potential of fungal cytochrome P450 enzymes [16]. Filamentous fungal genomes can contain 100–200 cytochrome P450 genes, representing a large potential reservoir of biocatalysts.

It has long been known that the efficiency of using certain filamentous fungi (e.g., *Aspergillus fumigatus*) for steroid biotransformations can be compromised by the production of unwanted side-product steroids [9, 17], and there is much interest in the scope to optimize and apply recombinant yeast as ‘microbial cell factories’ in order to achieve specific hydroxylations [10, 13, 16]. Recombinant strains of the fission yeast *Schizosaccharomyces pombe* have been employed for steroid bioconversions [18] including the 11α-hydroxylation of progesterone [15]. Designer strains of *Saccharomyces cerevisiae* that express mammalian steroid hydroxylases involved in xenobiotic metabolism (e.g., CYP3A4) have also been reported [19] that are capable of synthesizing hydrocortisone from glucose [20]. The scope of microbial biotechnology continues to expand and it is increasingly well recognized that the production of bio-based chemicals and biofuels from non-food carbohydrate feedstock using metabolically engineered yeast could offer a sustainable alternative to their petroleum-based production [21]. The application of recombinant yeast to assist the process development that is needed to overcome challenges in manufacturing and pharmaceutical industries has also been highlighted [22].

In the present study, we sought to develop a recombinant strain of *S. cerevisiae* that could hydroxylate progesterone at carbon 11α (Fig. 1) and that could be applied to the rural biorefinery concept [23] to achieve co-production of 11α-hydroxyprogesterone and bioethanol from grass juice feedstock [24]. We have previously shown that *S. pombe*, like *S. cerevisiae* cannot perform steroid hydroxylation in its native state [25] and it remains an attractive yeast host for heterologous protein expression. However, we chose to extend our work with *S. cerevisiae* [24, 26] because it is already employed in large-scale ethanol fermentations and because commercial and research ventures continue to yield more robust strains that are able to tolerate industrial conditions [21]. Grass juice sourced from *Lolium perenne* (ryegrass) represents one of several fractions of non-food plant biomass that are currently under investigation as feedstock for rural biorefinery and bio-based production processes in the United Kingdom [27–29]. It is rich in water-soluble carbohydrates (fructans, fructose, glucose, and sucrose) and its potential to support yeast growth and ethanol production has already been demonstrated [24, 26]. Therefore, producing a strain of *S. cerevisiae* that can produce both 11α-hydroxyprogesterone and ethanol using grass juice as carbon source can increase the financial yield from the bioconversion. As the global market for pharmaceutical steroid productions stands at around 10 billion USD [30] and the total cost of bioethanol is continually falling [31], using an integrative approach to coproduce a high-value platform chemical can increase the profitability of bioethanol production and agriculture.

Results from this study are the first to demonstrate functional expression of the 11α-steroid hydroxylase enzyme, documented for *A. ochraceus* [14], in *S. cerevisiae* grown on grass juice feedstock. This underscores the potential to diversify the scope of the rural biorefinery concept through application of yeast biotechnology, which represents an emergent and valuable asset for industries of the low-carbon and green economy.

## Results

### Recombinant yeast: progesterone bioconversion

**Fig. 2:**
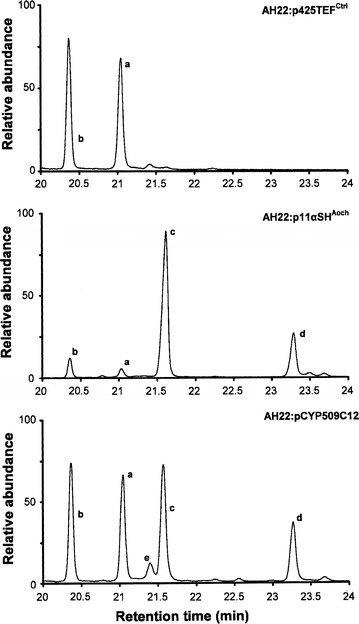
GC–MS chromatograms for progesterone substrate and hydroxylated metabolites extracted from steroid bioconversion experiments. See Table 1 and Fig. 3 for specific diagnostic and mass ion information. a Progesterone, b TMS-derivatized Progesterone, c diTMS-derivatized 11α-hydroxyprogesterone, d TMS-derivatized 11α-hydroxyprogesterone, e TMS-derivatized 6β-hydroxyprogesterone

**Fig. 3:**
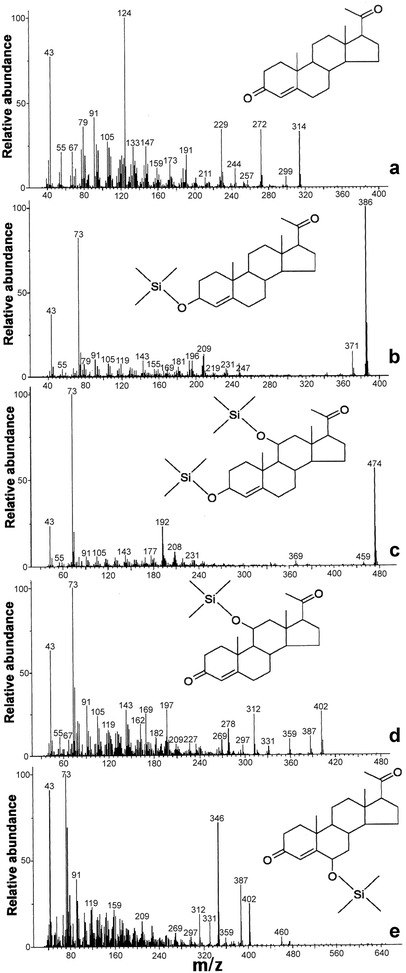
Fragmentation patterns (electron ionization) for progesterone substrate and hydroxylated metabolites. **a** Progesterone; **b** TMS-derivatized progesterone; **c** diTMS-derivatized 11α-hydroxyprogesterone; **d** TMS-derivatized 11α-hydroxyprogesterone; **e** TMS-derivatized 6β-hydroxyprogesterone. (TMS = trimethylsilyl derivatized)

Metabolite profiles for yeast transformants grown on YM+His with 300 μM progesterone indicate that the optimized gene sequences encode functional proteins that were constitutively expressed and conferred progesterone hydroxylating capabilities on AH22:p11α-SH^Aoch^ and AH22:pCYP509C12 yeast (Table 1; Fig. 2). During initial growth experiments with YM+His with 300 μM progesterone, almost total bioconversion of progesterone was achieved with AH22:p11α-SH^Aoch^ which produced ≥90% 11α-hydroxyprogesterone as the sole metabolite after 48 h of growth (Table 1). AH22:pCYP509C12 also produced 11α-hydroxyprogesterone, but with lower efficiency (47.61 ± 1.7%; *t*_48_ *h*) and lower purity as detectable quantities of 6β-hydroxyprogesterone (6.85 ± 1.7%; *t*_48_ *h*), identified from fragmentation spectra containing a prominent fragment ion at *m*/*z* 346 (Fig. 3e), were present in ethyl acetate extracts recovered from cell-free AH22:pCYP509C12 culture supernatant. Control (AH22:p425TEF^Ctrl^) yeast harboring empty p425TEF vector did not hydroxylate exogenous progesterone (Fig. 2), and only progesterone (Fig. 3a) or its trimethylsilyl (TMS) derivative (Fig. 3b) was detected in ethyl acetate extracts from AH22:p425TEF^Ctrl^ culture supernatant.

**Table 1:** GC–MS parameters and diagnostic ions for progesterone (Pg) substrate and hydroxylated (hydroxyPg) products

Peak	Gas chromatography–mass spectrometry: steroid identification				Steroid: % total (*t*_48_ *h*)		
	Steroid	RT (min)	[M]^+^	Fragment ions *m/z* (relative intensity)	AH22:p425TEF^Ctrl^	AH22:p11α-SH^Aoch^	AH22:pCYP509C12
a	Progesterone (Pg)	21.04	314	43 (999) 124 (887) 314 (701) 272 (430) 229 (362) 91 (329) 41 (314) 79 (313) 22 (238) 67 (234)	100	9.90 (±1.2)	45.54 (±3.1)
b	Pg + TMS (×1)	20.36	386	386 (999) 73 (820) 43 (365) 387 (311) 371 (145) 75 (141) 209 (132) 208 (118) 91 (101) 196 (97)			
c	11α-hydroxyPg + TMS (×2)	21.61	474	73 (999) 474 (559) 43 (230) 192 (227) 475 (219) 75 (198) 193 (126) 208 (85) 74 (84) 476 (80)	0	90.10 (±1.6)	47.61 (±1.7)
d	11α-hydroxyPg + TMS (×1)	23.29	402	73 (999) 43 (825) 75 (395) 402 (347) 91 (310) 312 (277) 197 (260) 143 (253) 169 (233) 105 (223)			
e	6β-hydroxyPg + TMS (×1)	21.40	402	73 (999) 43 (906) 346 (712) 75 (688) 91 (386) 387 (347) 79 (295) 93 (262) 402 (240) 119 (226)	0	0	6.85 (±1.7)
Maximum % bioconversion (ΣhydroxyPg)					0.00	91.70	57.86

Steroid (%) abundances are mean values from three replicates with associated standard deviations

*TMS* trimethylsilyl derivatized, *RT* retention time, *[M]* ^*+*^ mass ion

### Recombinant yeast: growth and ethanol fermentation

**Fig. 4:**
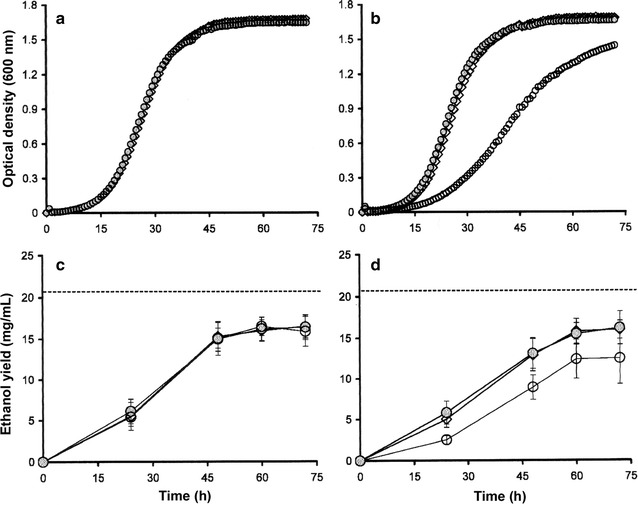
Growth profiles and ethanol yields. Growth profiles and ethanol yields for AH22:p11α-SH^Aoch^ (●), AH22:pCYP509C12 (◊), and AH22:p425TEF^Ctrl^ (◯) yeast grown on YM+His (**a**, **c**) or YM+His + 300 μM progesterone (**b**, **d**). Mean values of three replicates along with associated standard deviations are shown. The broken lines indicate maximum theoretical ethanol yield for 4% glucose

In studies undertaken using the Bioscreen C plate reader with starting (*t*_0_ *h*) inoculums of 5 × 10^5^ cells/mL and YM+His media (without progesterone supplementation), the growth parameters for AH22:p11α-SH^Aoch^, AH22:pCYP509C12, and AH22:p425TEF^Ctrl^ yeast were comparable (Table 2; Fig. 4). All constructs achieved similar final population densities (1.61 and 2.1 × 10^8^ cells/mL) and exhibited uniform doubling times (typically 4–5 h) at room temperature (20 ± 2 °C). There were no significant differences between the ethanol yields recorded for AH22:p11α-SH^Aoch^, AH22:pCYP509C12, and AH22:p425TEF^Ctrl^ grown on YM+His (ANOVA, *P* < 0.05, *n* = 3). All produced at least 16 mg/mL ethanol after 72 h fermentation; this equates to 78% of the theoretical maximum (20.4 mg/mL) for 4% glucose. In contrast, supplementation of YM+His with 300 µM progesterone had an inhibitory effect on the growth of AH22:p425TEF^Ctrl^ cultures which exhibited slower population doubling times (9–10 h), reached lower population densities (1.6 × 10^8^ cells/mL), and produced lower, more variable amounts of ethanol (12.5 ± 3.2 mg/mL) than those grown in the absence of progesterone (Table 2). Progesterone supplementation had no effect on the growth profiles or ethanol productivity of AH22:p11α-SH^Aoch^ or AH22:pCYP509C12 yeast (Table 2). In view of their bioconversion efficiency and 11α-hydroxyprogesterone specificity, only AH22:p11α-SH^Aoch^ yeast was selected for subsequent process optimization studies.

**Table 2:** Growth parameters and ethanol yields for recombinant yeast grown on 4% glcM^his^ ± Pg

Construct	ΔOD_600_ [*t*_48_ *h*]		Max. population doubling time (h)		Density: cells/mL [*t*_72_ *h*]		Ethanol: mg/mL [*t*_72_ *h*]	
	−Pg	+Pg	−Pg	+Pg	−Pg	+Pg	−Pg	+Pg
AH22:p425TEF^Ctrl^	1.61	0.98	4.6 (±0.5)	9.0 (±1.0)	2.1 × 10^8^	1.6 × 10^8^	15.9 (±1.4)	12.5 (±3.2)
AH22:p11α-SH^Aoch^	1.65	1.63	4.3 (±0.5)	4.5 (±0.5)	2.2 × 10^8^	2.1 × 10^8^	16.4 (±1.8)	16.2 (±2.0)
AH22:pCYP509C12	1.61	1.62	4.4 (±0.5)	4.6 (±0.5)	2.0 × 10^8^	2.1 × 10^8^	16.5 (±1.5)	16.0 (±1.1)

Mean values from three replicates are shown along with the associated standard deviations. Underlined values are significantly lower than those from comparator experiments (ANOVA, *P* < 0.05; *n* = 3)

### Two-step (bioconversion–fermentation) batch culture studies

**Fig. 5:**
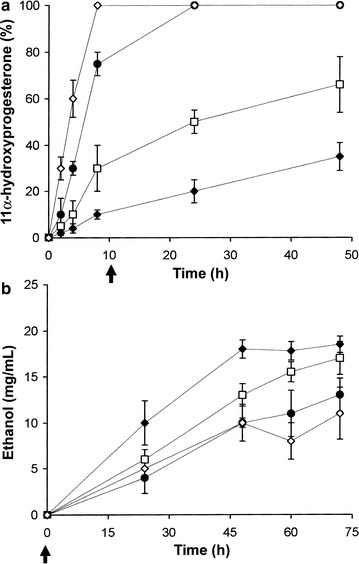
Optimized production of 11α-hydroxyprogesterone and ethanol using AH22:p11α-SH^Aoch^ yeast grown in batch culture with YM+His and 300 μM progesterone. Three shaker speeds, 30 (◆), 100 (□), 150 (●), and 200 (◊) rpm, were used. The arrow indicates the time point (*t*_10_ *h*) at which the speed of AH22:p11α-SH^Aoch^ cultures maintained at 200 rpm (optimal for progesterone bioconversion) **a** was either held at 200 rpm or decreased to 150, 100, or 30 rpm during the ethanol fermentation step (**b**). Mean values of three replicates along with associated standard deviations are shown

For AH22:p11α-SH^Aoch^ yeast (Fig. 5), complete progesterone bioconversion to 11α-hydroxyprogesterone was achieved after just 8 h when cultures were agitated continuously at 200 rpm at 20 °C. Following progesterone bioconversion (Step 1), decreasing the shaking regime from 200 to 30 rpm (at *t*_10_ *h*) was found to support optimal ethanol production of 18 ± 2 mg/mL (Fig. 5b) which was 88% of the theoretical maximum ethanol yield attainable with 4% glucose and 10% higher than that achieved in Bioscreen studies. In summary, the use of higher *t*_0_ *h* inoculums (1 × 10^8^ cells/mL) and vigorous culture agitation (200 rpm) facilitated progesterone bioconversion (Step 1). The higher cell densities (typically ≥3 × 10^8^ cells/mL) present by *t*_10_ *h* and lower agitation speeds (30 rpm) employed during fermentation (Step 2) resulted in higher ethanol yields.

### A biorefinery application for recombinant AH22:p11α-SH^Aoch^

**Fig. 6:**
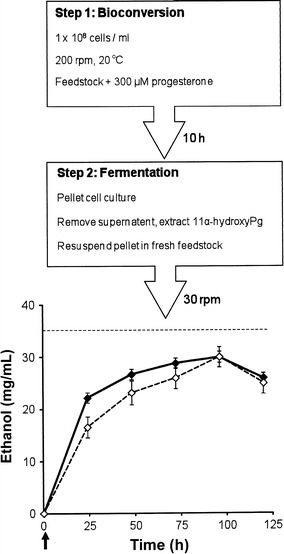
Preliminary biorefinery studies. These were performed using AH22:p11α-SH^Aoch^ yeast and synthetic 7% glucose YM+His (◆) or enzyme pretreated grass juice (◊) feedstock. The arrow indicates the time point (*t*_10_ *h*) at which AH22:p11α-SH^Aoch^ cultures were pelleted and resuspended in fresh feedstock. Broken line indicates maximum theoretical ethanol yield for 7% glucose. Mean values of three replicates along with associated standard deviations are shown

Complete bioconversion of progesterone to 11α-hydroxyprogesterone (Step 1) was achieved within 8 h when either grass juice with progesterone or 7% glucose YM+His with progesterone was used as feedstock for AH22:p11α-SH^Aoch^ yeast maintained at 20 ± 2 °C with vigorous shaking (200 rpm). As expected, ethyl acetate extraction of 11α-hydroxyprogesterone from the grass juice supernatant at the end (*t*_10_ *h*) of the bioconversion step (Fig. 6) resulted in the recovery of numerous phyto-chemicals that were identified using GC–MS (data not shown). The most abundant of these were 1-hexacosanol, phytol, α-tocopherol, 1-tetracosanol, 1-octacosanol, campesterol, stigmasterol, and β-sitosterol. Following resuspension of the AH22:p11α-SH^Aoch^ cell pellet in fresh feedstock (without progesterone supplementation) and after decreasing the culture agitation speed to 30 rpm for fermentation (Step 2), the maximum ethanol yields produced by AH22:p11α-SH^Aoch^ yeast on both grass juice and 7% glucose YM+His were 30–32 mg/mL. These ethanol yields exceed 85% of the theoretical maximum (35.7 mg/mL) for 7% glucose (Fig. 6). At the end of the studies undertaken using grass juice feedstock, screening of yeast cells plated onto selective YM+His agar at the end of both Step 1 (bioconversion) and Step 2 (fermentation) recovered a high percentage (typically ≥95%) of colony forming AH22:p11α-SH^Aoch^ yeast that had retained the plasmid.

### Progesterone bioconversion obtained with 11α-SH^Aoch^ integrated into *S. cerevisiae* clone

To establish whether expression of 11α-SH^Aoch^ using the p425 plasmid was comparable to integrating the gene, an integrated expression cassette, 11α-SH^Aoch^+K, was produced. Initial growth experiments in YM+His with 300 µM progesterone showed that AH22:11α-SH^Aoch^+K was able to achieve 58% conversion of progesterone to 11α-hydroxyprogesterone (174 µmol/L) after 48 h. Control AH22 cells did not hydroxylate progesterone as no steroid metabolites were detected upon extraction with ethyl acetate.

To investigate further whether 11α-SH^Aoch^ was able to metabolize higher concentrations of progesterone, the starting concentration was increased from 300 to 954 µM (0.3 g/L, in accordance with Žnidaršič et al. [32]) and the ratio of grass juice to YM, the inoculum size, and the incubation temperature were varied. Lowering the temperature from 30 to 24 °C increased the bioconversion of progesterone to 11α-hydroxyprogesterone from 42–50 to 54–62% after 48 h (Table 3). Diluting the grass juice with YM and using a lower inoculum only marginally affected the bioconversion rates. The maximum conversion rate obtained using AH22:11α-SH^Aoch^+K of 62% (591 µmol/L) was achieved after 48 h at 24 °C with a 1:1 mixture of grass juice and yeast minimal media (Table 3). This was a 3.4-fold increase in yield of 11α-hydroxyprogesterone compared to that obtained using 300 µM progesterone as substrate in YM+His and a 2.2-fold increase in yield compared to using clone AH22:p11α-SH^Aoch^ in YM+His after 48 h at 30 °C with 300 µM progesterone (Table 1).

**Table 3:** Bioconversion of progesterone to 11α-hydroxyprogesterone with AH22:11α-SH^Aoch^+K

Grass juice: yeast minimal	Inoculum (cells/mL)	Temperature (°C)	% product formed (µmol/L)
1:0	1 × 10^7^	30	47 (448)
1:0	1 × 10^7^	24	54 (515)
1:0	1 × 10^5^	30	44 (420)
1:0	1 × 10^5^	24	58 (553)
1:1	1 × 10^7^	30	44 (420)
1:1	1 × 10^7^	24	58 (553)
1:1	1 × 10^5^	30	44 (420)
1:1	1 × 10^5^	24	62 (591)
1:3	1 × 10^7^	30	42 (401)
1:3	1 × 10^7^	24	59 (563)
1:3	1 × 10^5^	30	50 (477)
1:3	1 × 10^5^	24	60 (572)

Progesterone bioconversion rates obtained using the integrated clone AH22:11α-SH^Aoch^+K with 0.3 mg/mL (954 µM) progesterone in growth media after 48 h

## Discussion

In situ hydroxylation of progesterone (Table 1) indicated both *A. ochraceus* 11α-SH and *R. oryzae* CYP509C12 were native steroid hydroxylating enzymes. AH22:p11α-SH^Aoch^ (Figs. 2, 3) produced 11α-hydroxyprogesterone as the sole product and AH22:pCYP509C12 produced a 7:1 mixture of 11α-hydroxyprogesterone and 6β-hydroxyprogesterone (Fig. 5e); *A. ochraceus* 11α-SH and *R. oryzae* CYP509C12 shared only 15% sequence identity (82 conserved amino acid residues) along the whole length of the two enzymes. However, NCBI BLAST2 analysis (http://blast.ncbi.nlm.nih.gov/Blast.cgi?PAGE_TYPE=BlastSearch&PROG_DEF=blastn&BLAST_PROG_DEF=megaBlast&BLAST_SPEC=blast2seq) identified 26% sequence identity (45 amino acid residues) in a 174 amino acid long region from the C-termini. The relatively low sequence identity between these two CYP enzymes suggests they belong to different CYP families. Native complementary DNA (cDNA) encoding the *A. ochraceus* 11α-SH has previously been co-expressed with cDNA for human oxidoreductase in *Spodoptera frugiperda* (Sf-9) insect cells [14]. Similarly, cDNA encoding the *R. oryzae* CYP509C12 has also been co-expressed with its native redox partner (*R. oryzae* NAD[P]H-dependent reductase) in recombinant fission yeast, *S. pombe*, which were also able to perform 11α- and 6β-steroid hydroxylation [15]. It is noteworthy that homology modeling and site-directed-mutagenesis studies have recently proved successful in redirecting the regio-selectivity of CYP106A2 (a mammalian P450 with 15β-hydroxylase and minor 11α-hydroxylase activity) from C15 to C11 [33]. The potential to bioengineer the specificity of fungal steroid hydroxylases (e.g., to remove the 6β-activity of CYP509C12) also exists and advances in this area now demand much better understanding of the catalytic and structural features of fungal steroid hydroxylases.

The recombinant AH22 (*S. cerevisiae*) whole-cell system reported in the present study extends the scope and application of the technological achievements of previous authors [14, 15] and demonstrates the constitutive expression of functional 11α-steroid hydroxylating enzymes in Brewer’s yeast. We chose to focus on the application of AH22:p11α-SH^Aoch^ yeast after preliminary metabolite studies as this biotransformation was highly specific and active. The maximum bioconversion initially achieved using AH22:p11α-SH^Aoch^ yeast and 300 µM progesterone was very high (90.9% 11α-hydroxyprogesterone or 273 µmol/L) and did not require heterologous electron donors to cytochrome P450 such as NADPH cytochrome P450 reductase (Table 1). The inhibitory effect of exogenous progesterone on the growth and ethanol yields of AH22:p425TEF^Ctrl^ (Table 2; Fig. 4) was consistent with the effect of steroid toxicity on *S. cerevisiae* [34]. The growth rates and ethanol yields for AH22:p11α-SH^Aoch^ and AH22:pCYP509C12 grown on YM+His with or without progesterone were comparable suggesting that constitutive expression of *A. ochraceus* 11α-SH and *R. oryzae* CYP509C12 served a protective role in recombinant AH22:p11α-SH^Aoch^ and AH22:pCYP509C12 yeast. The ability of *Aspergillus* and *Rhizopus* spp. to hydroxylate and excrete steroids represents one mechanism through which filamentous species are believed to detoxify and so persist when exposed to steroids or similar molecules [35].

By increasing the starting inocula from 5 × 10^5^ to 1 × 10^8^ cells/mL in optimization studies undertaken with AH22:p11α-SH^Aoch^ yeast (Fig. 5) and through initial application of vigorous culture agitation (Step 1: bioconversion, 200 rpm), we achieved 100% conversion of progesterone to 11α-hydroxyprogesterone in just 8 h. Here, it is likely that increased oxygenation of the culture medium favored the growth of AH22:p11α-SH^Aoch^ yeast and facilitated the catalytic activity of constitutively expressed *A. ochraceus* 11α-SH monooxygenase. During fermentation (Step 2), the presence of higher cell densities (≥3 × 10^8^ cells/mL) and the reduction of culture agitation speeds from 200 to 30 rpm (Fig. 5) improved the ethanol yields from 78% (Bioscreen studies) to 88%. These results reflect a reduction in the amount of glucose used for growth under semi-anaerobic conditions and an increase in the fermentation of glucose to ethanol; this is further evidenced by the lower ethanol yields recorded during fermentation (Step 2) regimes (100, 150 and 200 rpm) that increased the oxygenation of AH22:p11α-SH^Aoch^ cultures (Fig. 5).

Agriculturally sourced grass juice feedstock was amenable to the two-step bioconversion–fermentation process developed using AH22:p11α-SH^Aoch^ yeast (Fig. 6) presenting bioengineering opportunities within biorefinery settings in temperate regions [27–29]. Firstly, it is significant that high product (11α-hydroxyprogesterone and ethanol) yields were achievable using grass juice feedstock at room temperature (20 ± 2 °C). On an industrial scale, this could help reduce the need for energy-intensive heating and cooling regimes. Secondly, the solvent extraction of 11α-hydroxyprogesterone product from the grass juice/progesterone feedstock resulted in the recovery of numerous phyto-chemicals including 1-hexacosanol, 1-tetracosanol, 1-octacosanol (all commercially relevant fatty alcohols), phytol and α-tocopherol (both vitamin E precursors with applications in cosmetics, detergents, and as antioxidants), and campesterol, stigmasterol, and β-sitosterol (plant sterols with dietary health benefits). Commercial interest in high-value phyto-chemicals [23] strengthens the premise for using grass juice and other plant-based substrates as feedstock for progesterone bioconversion and the development of technologies that can optimize the recovery (extraction, separation, and purification) of multiple desirable products is an emergent and necessary area of research [36].

Further efforts towards yield optimization were focused on the production of the high-value platform chemical 11α-hydroxyprogesterone by the 11α-steroid hydroxylase enzyme from *A. ochraceus*. The enzyme was integrated into the *S. cerevisiae* genome to facilitate clone stability (no risk of losing the enzyme through plasmid loss over time). Increasing the progesterone concentration from 300 to 954 µM and reducing the incubation temperature from 30 to 24 °C resulted in 2.2-fold greater bioconversion to 11α-hydroxyprogesterone than obtained using the yeast strain containing the steroid hydroxylase on a plasmid (AH22:p11α-SH^Aoch^). The increased yield of product at lower temperature reduces the external energy requirements and therefore increases the cost effectiveness of the bioconversion of progesterone to 11α-hydroxyprogesterone. *A. ochraceus* steroid 11α-hydroxylase was also regiospecific, only hydroxylating progesterone at the 11α- position, unlike *R. oryzae* steroid hydroxylase which hydroxylated progesterone at two positions (11α- and 6β-). Therefore, the use of *A. ochraceus* steroid 11α-hydroxylase would be the more economical as further expense to separate hydroxyprogesterone isomers would not be required. However, further optimization is still required before process scale-up can take place [22]. High ethanol yields (14.9% v/v) have recently been achieved using pure inulin and soybean feedstock and an engineered yeast strain (*Saccharomyces* sp. W0) that expresses the inulinase gene from *Pichia guilliermondii* [37]. The use of grass juice feedstock extracted from high sugar varieties of ryegrass [29] and/or alternative (e.g., flocculating) host strains of *S. cerevisiae* that exhibit enhanced ethanol production [38] remains to be investigated. The influence of other factors including nutrient supplements, temperature, pH stress, and culture volume on ethanol yields from grass juice warrants investigation and the use of chemostat and bioreactor platforms represents the next step for research development [39].

In light of the successful bioconversion of progesterone to 11α-hydroxyprogesterone by AH22:11α-SH^Aoch^+K, stable, genome-integrated designer yeasts that are able to exploit specific feedstocks for the co-production of high-value products provide an important avenue for commercial biotransformations of chemicals. For example, yeasts could be utilized for the production of dietary mycoprotein, other myco-products (e.g., sterols, fatty acids, minerals, and B-complex vitamins), and specific platform chemicals (e.g., vitamin precursors, NADPH, and other cofactors required for growth) and opportunities for the development of industrial scale biotechnology exist here.

## Conclusions

This study explored the potential for biotransformation for co-products to add value to a biofuel production process using a novel feedstock. Production of a key intermediate of corticosteroid production using fungal enzymes was demonstrated, a biotransformation of historical interest among biotransformations. Further biotransformations can be envisaged using the yeast biomass involved in bioethanol production as well as recovery of useful cellular components of yeast from the fermentation.

## Methods

### Sequence optimization

Genes encoding *A. ochraceus* 11α-steroid hydroxylase (11α-SH^Aoch^) (Genbank accession number ABH71415.1) [14] and *R. oryzae* CYP509C12 (Genbank accession number RO3G_05077.1) [15] were designed with codon bias optimized for *S. cerevisiae* and the following modifications: (i) an AAA triplet (Kozak sequence) upstream of ATG initiation codons, (ii) external *Not*I single restriction sites (for the excision of whole fragments), and (iii) flanking *Bam*HI and *Xho*I single restriction sites for the double-digest and direct ligation of genes into p425TEF (Dualsystems Biotech), a shuttle vector [ampicillin selection (bacteria) and *LEU2* auxotrophic marker (yeast)], that enables constitutive expression driven by the transcription elongation factor (TEF1) promoter [40]. Both genes were synthesized commercially (Eurofins MWG Operon) and supplied in pEX-K vector (kanamycin selection) for maintenance in *Escherichia coli*.

### Plasmid construction and recombinant yeast

Optimized genes for *A. ochraceus* and *R. oryzae* were excised from pEX-K and ligated into pre-cut (*Bam*HI, *Xho*I) p425TEF vector. The resulting plasmids (p11α-SH^Aoch^ and pCYP509C12, respectively) were transformed into *E. coli* strain DH5α (Novagen) that were selected and maintained at 37 °C using Luria–Bertani medium supplemented with sodium ampicillin (100 mg/L). Purified p11α-SH^Aoch^ and pCYP509C12 plasmid DNA were isolated from *E. coli* cultures (Wizard Plus SV Minipreps, Promega) and transformed into *S. cerevisiae* strain AH22 (*MAT*a, *leu2*-*3*, *112 his4*-*519 can1*) via electroporation to yield the experimental constructs AH22:p11α-SH^Aoch^ and AH22:pCYP509C12. A control construct (AH22:p425TEF^Ctrl^) was created by transforming AH22 with p425TEF vector. All AH22 yeast transformants were selected and maintained at 30 °C using glucose-based yeast minimal (YM+His) plates containing (w/v) 2% agarose, 1.34% yeast nitrogen base without amino acids, 4% glucose, and l-histidine (100 mg/L).

### Progesterone conversion

AH22:p11α-SH^Aoch^, AH22:pCYP509C12, and AH22:p425TEF^Ctrl^ transformants were screened for their ability to convert progesterone into hydroxylated products by inoculating 60 mL volumes of YM+His + 300 µM progesterone with single colonies (*n* = 3) taken directly from transformation plates. Cells were grown in 100-mL flasks (30 ± 2 °C, 200 rpm) and harvested by centrifugation (4000 rpm, 5 min) after 48 h. Residual progesterone substrate and hydroxyprogesterone products were extracted from 5 mL volumes of cell-free culture supernatant with 3 × 2 mL volumes of ethyl acetate which were pooled and evaporated to dryness using a centrifugal evaporator (Heto Maxi dry plus). Extracts were stored at −20 °C prior to further analysis. Fresh 100 mM progesterone stocks (31.4 mg progesterone dissolved in 1 mL DMSO) were used to prepare YM+His + 300 μM progesterone for all experimental work.

### Gas chromatography–mass spectrometry (GC–MS) metabolite analysis

Prior to GC–MS analysis, steroid extracts were heated (70 °C, 10 min) to ensure complete dryness and derivatized using 100 µL *N*,*O*-bis(trimethylsilyl)trifluoroacetamide-trimethylchlorosilane (BSTFA-TMCS [99:1]) and 100 µL anhydrous pyridine heated at 70 °C for 2 h [41]. Trimethylsilyl (TMS)-derivatized sterols were analyzed using a 7890A GC–MS system (Agilent Technologies) with a DB-5MS fused silica column (30 m × 0.25 mm × 0.25 µm film thickness; J&W Scientific). The oven temperature was initially held at 70 °C for 4 min, and then increased at 25 °C/min to a final temperature of 280 °C, which was held for a further 25 min. Samples were analyzed in splitless mode (1 µL injection volume) using helium carrier gas, electron ionization (ion source temperature of 150 °C), and mass scanning from *m/z* 40 to 850 [41]. GC–MS data files were analyzed using MSD Enhanced ChemStation software (Agilent Technologies Inc.) to determine metabolite profiles for AH22:p11α-SH^Aoch^, AH22:pCYP509C12, and AH22:p425TEF^Ctrl^ and for derivation of integrated peak areas. Progesterone and hydroxyprogesterone metabolites were identified with reference to retention times and mass fragmentation patterns for known standards. Phyto-chemicals that were co-extracted from grass juice feedstock in ‘Biorefinery pilot studies’ were identified using the NIST Mass Spectral Search Program (NIST/EPA/NIH Mass Spectral Library, Version 2.0 g, May 2011) and with reference to diagnostic fragmentation spectra.

### Growth and ethanol production

Growth rate and ethanol productivity comparisons for AH22:p11α-SH^Aoch^, AH22:pCYP509C12, and AH22:p425TEF^Ctrl^ grown on YM+His with 300 μM progesterone were undertaken in 100-well honeycomb microplates using a Bioscreen C (Oy Growth Curves Ab Ltd., Finland) as previously described [26]. Briefly, uniform starting (*t*_0_ *h*) culture densities were achieved by resuspending single colonies in YM+His (with or without 300 μM progesterone) and diluting to achieve 5 × 10^5^ cells/mL. Starting cultures were vortexed and dispensed into Bioscreen wells (3 × 300 µL replicates per colony suspension). All experiments were incubated at 20 ± 2 °C in the Bioscreen (no shaking regime) for 72 h, with optical density (at 600 nm) readings taken every 45 min. Growth data were exported from the Bioscreen in ASCII format prior to analysis using Excel (Microsoft Office 2003).

### Growth parameters

In addition to manual (Neubauer haemocytometer) cell counts, ΔOD_600_ values (derived by subtracting the starting optical density from the final reading) were recorded. Maximum doubling times were estimated by dividing the natural logarithm of 2 by the fastest population growth rates (*μ*) derived using linear trend lines (*y* = *mx* + *c*, where *y* = vertical axis coordinate; *m* = gradient; *x* = horizontal axis coordinate and *c* = *y* axis intercept) fitted to log-transformed OD data. For ethanol analyses, Bioscreen measurements were suspended at specific time points (*t*_0_ *h*, *t*_24_ *h*, *t*_48_ *h*, and *t*_72_ *h*) and a 10 μL volume of culture supernatant removed from representative experimental wells.

### Ethanol content

Ethanol content was determined using a spectrophotometric ethanol assay kit (K-ETOH 11/06; Megazyme Ltd.) according to manufacturer’s instructions. All samples were diluted 1000-fold with distilled water prior to analysis. Ethanol yields were calculated as a percentage of the maximal theoretical yield for glucose (0.51 g/g).

### Two-step batch culture studies: optimization of shaking regime

Two-step (bioconversion–fermentation) batch culture studies were performed at 20 ± 2 °C with AH22:p11α-SH^Aoch^ yeast grown in 100-mL conical flasks containing 60 mL volumes of YM+His with 300 μM progesterone. Step 1 (progesterone bioconversion) experiments were initiated with *t*_0_ *h* inoculums of 1 × 10^8^ cells/mL that were agitated at speeds of 30, 100, 150, or 200 rpm for 48 h. One milliliter volumes were aseptically removed from experimental flasks at *t*_2_ *h*, *t*_4_ *h*, *t*_8_ *h*, *t*_24_ *h*, and *t*_48_ *h* in order to determine % conversion of progesterone to 11α-hydroxyprogesterone. Step 2 (ethanol fermentation) optimization was pursued using AH22:p11α-SH^Aoch^ that had been maintained (throughout Step 1) at 200 rpm on YM+His with 300 μM progesterone. At *t*_10_ *h,* the shaking speed of these cultures was decreased to 150, 100, and 30 rpm or held at 200 rpm for a further 75 h. One milliliter volumes were removed from fermentation cultures for the determination of ethanol content at *t*_24_ *h*, *t*_48_ *h*, *t*_60_ *h,* and *t*_72_ *h*. The theoretical maximum ethanol yield for 4% (40 mg/mL) glucose (assuming 0.51 g/g) was calculated to be 20.4 mg/mL.

### Biorefinery studies using grass juice feedstock

Biorefinery studies were performed using AH22:p11α-SH^Aoch^ yeast and enzyme (*Lactobacillus paracasei* β-fructosidase) pretreated grass juice comprising 70 mg/mL monosaccharide [24] or synthetic 7% glucose YM+His (containing 70 mg/mL glucose equivalent) as feedstock. Grass juice was extracted from ryegrass *L. perenne* supplied by the Institute of Biological, Environmental Research and Rural Sciences (IBERS, Aberystwyth, UK), screened to remove large particulates, autoclaved (121 °C, 30 min), and pretreated with purified *L. paracasei* β-fructosidase [24] prior to use as a growth and fermentation substrate. Step 1 (progesterone bioconversion) was initiated with 60 mL volumes of grass juice + 300 μM progesterone or 7% glucose YM+His + 300 μM progesterone and *t*_0_ *h* inoculums of 1 × 10^8^ cells/mL that were maintained in 100-mL conical flasks at 20 ± 2 °C and agitated at 200 rpm. After 10 h, AH22:p11α-SH^Aoch^ cultures were harvested by centrifugation (4000 rpm, 5 min) and metabolites in the cell-free culture supernatant were extracted using ethyl acetate for GC–MS analysis. Cell pellets were then resuspended in 60 mL volumes of fresh grass juice or 7% glucose YM+His (without progesterone) and maintained for a further 120 h at 20 ± 2 °C and at a shaking speed of 30 rpm. One milliliter volumes were removed at *t*_24_ *h*, *t*_48_ *h*, *t*_72_ *h*, *t*_96_ *h*, and *t*_120_ *h* for the determination of ethanol content. The theoretical maximum ethanol yield for 7% (70 mg/mL) glucose (assuming 0.51 g/g) was calculated to be 35.7 mg/mL. For studies using grass juice feedstock, colony screening of AH22:p11α-SH^Aoch^ cells (100 µL of a 1 × 10^3^ cells/mL dilution) plated onto selective YM+His agar was performed to calculate the recovery (%) of yeast that had retained the p11α-SH^Aoch^ plasmid at the end of Step 1 (bioconversion; *t*_10_ *h*) and Step 2 (fermentation; *t*_125_ *h*).

### Integration of 11α-SH^Aoch^ into the genome of *S. cerevisiae*

The *11α*-*SH* ^*Aoch*^ gene was synthesized (Eurofins MWG Operon) with optimization for *S. cerevisiae* and supplied in the vector pBSII SK(+). Forward and reverse PCR primers were prepared to produce a cassette for the transformation of *S. cerevisiae* AH22 cells. Primers were designed to include PCR priming sites for ends of the gene (how many bases?), 50-bp complementary sequences upstream of the *Ura3* gene and downstream of the *Ura3* gene, *Sal*I/*Spe*I restriction sites for insertion of the KanMX marker, and *Not*I sites for excision from the plasmid. The PCR cassette was inserted into pBSII SK(+) at the *Not*I site (chosen as it had been altered to remove the *Sal*I and *Spe*I sites) producing pBSII SK(+):11α-SH^Aoch^+K. Construct integrity was checked by DNA sequencing. A KanMX cassette was cut out of pAH3 vector using *Sal*I–*Spe*I digest and inserted into the *Sal*I/*Spe*I site of pBSII SK(+):11α-SH^Aoch^+K to give the final transformation cassette. pBSII SK(+):11α-SH^Aoch^+K was digested with *Not*I prior to transformation into chemically competent AH22 cells. Positive transformants were selected using geneticin in YPD and were checked by sequencing PCR products produced when using primers located upstream and downstream of the *Ura3* site, internal 11α-SH^Aoch^ gene primers, and KanMx primers.

The ability of AH22:11α-SH^Aoch^+K to hydroxylate progesterone was assessed using the methodology described above, except bioconversion was also measure when 954 µM (0.3 g/L) of progesterone was added to YM.

## Abbreviations

BSTFA: *N*,*O*-bis(trimethylsilyl)trifluoroacetamide
DMSO: dimethylsulfoxide
EtOH: ethyl alcohol
GC–MS: gas chromatography–mass spectrometry
TMCS: trimethylchlorosilane
TMS: trimethylsilanyl
